# BAP31 Modulates Mitochondrial Homeostasis Through PINK1/Parkin Pathway in MPTP Parkinsonism Mouse Models

**DOI:** 10.3390/cells15020137

**Published:** 2026-01-12

**Authors:** Wanting Zhang, Shihao Meng, Zhenzhen Hao, Xiaoshuang Zhu, Lingwei Cao, Qing Yuan, Bing Wang

**Affiliations:** 1 College of Life Science and Health, Northeastern University, Shenyang 110169, China; 2 Jiangsu Key Laboratory of Marine Pharmaceutical Compound Screening, Jiangsu Ocean University, Lianyungang 222005, China

**Keywords:** Parkinson’s disease, BAP31, mitochondrial homeostasis, PINK1–Parkin pathway, EN1

## Abstract

Parkinson’s disease (PD) is a neurodegenerative disorder characterized by age-dependent degeneration of dopaminergic neurons in the substantia nigra, a process mediated by α-synuclein aggregation, mitochondrial dysfunction, and impaired proteostasis. While BAP31—an endoplasmic reticulum protein critical for protein trafficking and degradation—has been implicated in neuronal processes, its role in PD pathogenesis remains poorly understood. To investigate the impact of BAP31 deficiency on PD progression, we generated dopamine neuron-specific BAP31 conditional knockout with DAT-Cre (cKO) mice (Slc6a3cre-BAP31^fl/fl^) and subjected them to MPTP-lesioned Parkinsonian models. Compared to BAP31^fl/fl^ controls, Slc6a3cre-BAP31^fl/fl^ mice exhibited exacerbated motor deficits following MPTP treatment, including impaired rotarod performance, reduced balance beam traversal time, and diminished climbing and voluntary motor capacity abilities. BAP31 conditional deletion showed no baseline phenotype, with deficits emerging only after MPTP. Our results indicate that these behavioral impairments correlated with neuropathological hallmarks: decreased NeuN neuronal counts, elevated GFAP astrogliosis, reduced tyrosine hydroxylase levels in the substantia nigra, and aggravated dopaminergic neurodegeneration. Mechanistically, BAP31 deficiency disrupted mitochondrial homeostasis by suppressing the PINK1–Parkin mitophagy pathway. Further analysis revealed that BAP31 regulates PINK1 transcription via the transcription factor Engrailed Homeobox 1. Collectively, our findings identify BAP31 as a neuroprotective modulator that mitigates PD-associated motor dysfunction by preserving mitochondrial stability, underscoring its therapeutic potential as a target for neurodegenerative disorders.

## 1. Introduction

Parkinson’s disease (PD) has emerged as a global health challenge, with its prevalence tripling over the past three decades due to aging populations and environmental triggers [1,2]. This progressive neurodegenerative disorder is characterized not only by classic motor symptoms, including resting tremors, bradykinesia, and postural instability, but also by debilitating non-motor manifestations such as REM sleep behavior disorder, autonomic dysfunction, and cognitive decline, all of which severely compromise quality of life [3,4]. Mounting evidence highlights a complex interplay between genetic susceptibility and environmental risk factors, which collectively disrupt proteostasis networks and drive disease progression [5,6]. Central to PD pathogenesis is the misfolding and aggregation of α-synuclein into Lewy bodies, which propagate via prion-like mechanisms across neural circuits, impair synaptic vesicle recycling, and trigger neuroinflammation [7,8]. Furthermore, recent advances implicate mitochondrial bioenergetic failure, marked by disrupted electron transport chain activity and ROS overproduction, and lysosomal–autophagic dysfunction in the selective vulnerability of dopaminergic neurons [9,10]. These insights have spurred interest in targeting mitochondrial quality control pathways, particularly the PINK1/Parkin-mediated mitophagy axis, as a therapeutic strategy to halt neurodegeneration [11].

BAP31, a ubiquitously expressed endoplasmic reticulum (ER) membrane protein, serves as a multifunctional regulator of cellular processes, including protein trafficking, apoptotic signaling, and ER-mitochondrial calcium crosstalk [12,13]. Its pronounced expression in the brain underscores potential roles in neurological disorders, particularly neurodegenerative diseases such as Alzheimer’s disease (AD), PD, and amyotrophic lateral sclerosis/frontotemporal dementia (ALS/FTD) [14,15], where dysregulated ER-mitochondria communication is increasingly implicated in pathogenesis. While our prior work demonstrated its neuroprotective function in AD by stabilizing reticulon 3 (RTN3) to suppress amyloid-β (Aβ) production [16], BAP31’s contribution to PD-specific mechanisms—such as dopaminergic neuron survival, α-synuclein aggregation, and mitochondrial dynamics—remains poorly characterized. Elucidating how BAP31 modulates these PD-relevant pathways could unveil novel therapeutic strategies targeting ER–mitochondria interactions, which are critical for maintaining neuronal homeostasis [17,18].

PTEN-induced kinase 1 (PINK1), a mitochondrial serine/threonine kinase, plays a pivotal role in PD pathogenesis. Loss-of-function mutations in PINK1 are linked to early-onset PD [19], primarily due to its essential role in mitochondrial quality control and neuronal survival. PINK1 acts as a sensor of mitochondrial damage, initiating Parkin-mediated mitophagy to eliminate dysfunctional mitochondria and prevent the accumulation of cytotoxic reactive oxygen species (ROS) [20,21]. Under oxidative stress, PINK1 deficiency disrupts this protective mechanism, leading to persistent mitochondrial damage, exacerbated ROS production, impaired energy metabolism, and ultimately dopaminergic neurodegeneration [22,23]. Beyond mitophagy, PINK1 regulates mitochondrial fission/fusion dynamics and calcium homeostasis to maintain mitochondrial network stability [24,25]. It also coordinates with endoplasmic reticulum (ER)-mitochondria interaction proteins to orchestrate integrated stress responses, highlighting its potential as a therapeutic target for mitochondrial rescue strategies in PD [26,27]. Given PINK1’s central role in neurodegenerative disorders such as PD, transcriptional regulators like the Engrailed (EN) family factors (EN1 and EN2) are of particular interest. Given PINK1’s central role in neurodegenerative disorders such as PD, investigating EN1-mediated control of PINK1 may unveil novel mechanistic insights into disease pathogenesis. For instance, dysregulation of the EN1-PINK1 axis could impair mitochondrial surveillance, exacerbating neuronal vulnerability to proteotoxic and oxidative insults—a hypothesis with broad implications for therapeutic development [28,29].

## 2. Materials and Methods

### 2.1. BAP31 Conditional Knockout with Slc6a3-Cre Mice

To generate dopamine neuron-specific BAP31 conditional knockout with DAT-Cre (cKO) mice, we first designed a targeting vector containing a BAP31 intronic sequence flanked by two loxP sites. The neomycin resistance (NeoR) cassette, used for positive selection, was inserted between two FRT sites (Flp recombinase recognition targets) to allow subsequent removal [30]. The linearized targeting vector was electroporated into C57BL/6 embryonic stem (ES) cells, followed by G418 selection to identify homologous recombinant clones. Correctly targeted ES cells were microinjected into blastocysts to generate chimeric mice, which were then bred to C57BL/6 females to obtain germline-transmitted BAP31^fl/fl^ offspring. To achieve dopaminergic neuron-specific deletion, BAP31^fl/fl^ mice were crossed with Slc6a3-Cre transgenic mice expressing Cre recombinase under the dopamine transporter (DAT) promoter. By crossing BAP31^fl/fl^ mice with Slc6a3-Cre mice to obtain the F1 generation, and then crossing the F1 male mice with BAP31^fl/fl^ female mice, the experimental mice (Slc6a3cre-BAP31^fl/fl^) and the control group mice (BAP31^fl/fl^) were obtained. Experimental (Slc6a3cre-BAP31^fl/fl^) and control (BAP31^fl/fl^) animals were littermates, thereby minimizing potential confounding effects of genetic variability on phenotypic outcomes. All experiments utilized 6-month-old male and female mice to minimize age-related variability [31]. Behavioral data from male and female mice were pooled for analysis, as preliminary assessment indicated no significant main effect of sex or sex-by-genotype interaction on the core behavioral outcomes measured in this MPTP model. All animal cohorts involved in comparative analyses (e.g., Slc6a3cre-BAP31^fl/fl^ vs. BAP31^fl/fl^ littermates under MPTP or saline treatment) were bred, behaviorally tested, and sacrificed for tissue collection within the same seasonal period to control for potential confounding effects of endogenous seasonal biological rhythms on neurochemistry and behavior. Animal protocols were approved by the Animal Care and Use Committee of Northeastern University and conducted in accordance with the National Institutes of Health Guide for the Care and Use of Laboratory Animals. Mice were housed under standard conditions (12-hr light/dark cycle, 22 °C) with ad libitum access to food and water.

### 2.2. SH-SY5Y Cells

Human neuroblastoma (SH-SY5Y) cells were cultured in Dulbecco’s Modified Eagle Medium/Nutrient Mixture F-12 (DMEM/F12) supplemented with 10% fetal bovine serum (FBS), 1% penicillin–streptomycin, and 2 mM L-glutamine, and maintained at 37 °C in a humidified incubator with 5% CO_2_. For transient BAP31 knockdown, SH-SY5Y cells were transfected with BAP31-specific siRNA (siBAP31) or scrambled control siRNA (siCtrl) using Lipofectamine 3000 (Thermo Fisher Scientific, Waltham, MA, USA, Cat. L3000008) according to the manufacturer’s protocol. Briefly, siRNA-Lipofectamine complexes were prepared in Opti-MEM serum-free medium and incubated with cells for 6 h, followed by replacement with complete medium. Knockdown efficiency was validated 48 h post-transfection by Western blotting.

### 2.3. Brain Slice Processing

Following behavioral testing, mice were deeply anesthetized and transcardially perfused. The perfusion needle was inserted into the left ventricular apex and advanced through the aortic arch. Systemic perfusion was initiated with ice-cold phosphate-buffered saline (PBS) to clear circulating blood, followed by fixation with freshly prepared 4% paraformaldehyde (PFA) in 0.1 M phosphate buffer (pH 7.3). After complete fixation, brains were carefully extracted and post-fixed in the same fixative for 48 h at 4 °C. Coronal blocks of 3 mm thickness were prepared using a rodent brain matrix. Tissue processing included dehydration through a graded ethanol series (70–100%), clearing in xylene, and paraffin embedding using standard histological protocols. Subsequently, 5 μm thick serial sections were cut on a rotary microtome and mounted on gelatin-coated slides for subsequent histological analyses.

### 2.4. TH Staining

The total number of TH^+^ neurons and Nissl-stained neurons was quantified by unbiased stereology using the optical fractionator method, as previously described. TH (#ab137869, 1:1000, Abcam, Cambridge, MA, USA).

### 2.5. Isolation and Cultivation of Primary DA Neurons

The entire procedure for the isolation and identification of primary dopaminergic (DA) neurons involves dissecting the ventral mesencephalon from E14-E16 rodent embryos, digesting the tissue with trypsin or papain, and triturating to create a single-cell suspension; after centrifugation, cells are resuspended in Neurobasal-A or DMEM/F-12 medium supplemented with B-27, growth factors, and plated on poly-D-lysine/laminin-coated surfaces at a density of 5.0 × 10^5^ cells/mL; cultures are maintained at 37 °C with 5% CO_2_, with medium changes every 2–3 days and optional cytosine arabinoside treatment to suppress glial growth; between days 7–14 in vitro, cells are fixed for immunostaining using antibodies against tyrosine hydroxylase (TH) for DA neuron identification and β-tubulin III or MAP2 as pan-neuronal markers, followed by fluorescence visualization and quantification of TH-positive cells relative to total neurons.

### 2.6. Western Blotting (WB)

The protocol used for WB analysis was as reported previously. Briefly, brain tissues and cells were homogenized in radioimmunoprecipitation assay lysis buffer. A 30-μg aliquot of protein from each sample was separated using sodium dodecyl sulfate-polyacrylamide gel electrophoresis (SDS-PAGE; 10–12%) and transferred onto PVDF membranes (IPVH00010, Millipore, Billerica, MA, USA), which were blocked with 10% nonfat dry milk in Tris-buffered saline with Tween-20 (#T104863, Aladdin, Shanghai, China). Membranes were probed with primary antibodies overnight at 4 °C and incubated with horseradish peroxidase-conjugated goat anti-mouse (#31430, Invitrogen, Life Technologies Corporation, Carlsbad, CA, USA) or rabbit (#31460, Invitrogen, Life Technologies Corporation, Carlsbad, CA, USA) IgG secondary antibodies (1:2000). Antibodies to BAP31 (#12226-1-AP, 1:1000, Proteintech Group, Chicago, IL, USA), TH (#ab6211, 1:1000, Abcam, Cambridge, MA, USA), PINK1 (Santa Cruz;sc-517353, 1:1000, Signalway Antibody, College Park, MD, USA), BAP31 (11200-1-AP, 1:1000, Proteintech), PARK7 (11681-1-AP, 1:1000, Proteintech), PARK2 (14060-1-AP,1:1000, Proteintech), SP1 (WL02251, 1:1000, Wanlei Biotechnology, Shanghai, China), EN1 (ab108598, 1:1000, Abcam), and MFF (12186-1-AP, Proteintech) were used. Detection was performed using an ImageQuant LAS 4000 mini luminescent image analyzer (Cytiva, Uppsala, Sweden). Band intensity was quantified using ImageJ software 1.53t (NIH, Bethesda, MD, USA).

### 2.7. Immunohistochemistry (IHC)

For IHC staining, brain sections were dehydrated in a graded series of alcohol solutions after post-fixation. As previously described, brain tissue encompassing the entire substantia nigra pars compacta was sectioned into 30 μm slices using a cryostat (M1950-1-0-0-1-1, Leica Microsystems, Nussloch, Germany), incubated with 0.3% Triton X-100 in phosphate-buffered saline (PBS) for 15 min, and blocked with 5% goat serum (#16210-064, Invitrogen, Life Technologies Corporation, Carlsbad, CA, USA) for 1 h at 24 ± 2 °C. Subsequently, the sections were incubated with specific primary antibodies at 4 °C overnight. Antibodies against BAP31 (11200-1-AP, 1:1000, Proteintech Group, Chicago, IL, USA), NeuN (#ab177487, 1:2000, Abcam, Cambridge, MA, USA), and GFAP (#MAB360, 1:800, Millipore, Billerica, MA, USA) were used.

### 2.8. Immunofluorescence

For immunofluorescence, the sections were washed and incubated for 1 h with Alexa Fluor 488-conjugated donkey anti-mouse IgG (#A21202, 1:800, Invitrogen, Life Technologies Corporation, Carlsbad, CA, USA) or Alexa Fluor 555-conjugated goat anti-Rat IgG (#A21432, 1:800, Invitrogen, Life Technologies Corporation, Carlsbad, CA, USA). After a final wash with PBS, the sections were mounted onto glass slides, and ProLong gold anti-fade reagent containing DAPI (#P36931, Invitrogen, Life Technologies Corporation, Carlsbad, CA, USA) was applied to visualize nuclei. For IHC, the slides were incubated with streptavidin-HRP (#31430, 31460, 31402, 1:1000, Invitrogen, Life Technologies Corporation, Carlsbad, CA, USA) for 40 min and stained with DAB (#GK500705, Gene Tech, Shanghai, China).

### 2.9. HPLC Analysis of DOPAC and HVA

Striatal tissue samples were homogenized in 0.1 M perchloric acid and centrifuged at 14,000× *g* for 20 min. The resulting supernatant was filtered through a 0.22 μm membrane before injection into a high-performance liquid chromatography system equipped with a fluorescence detector (HPLC-FD). Separation was performed using a Diamonsil C18 column (250 mm × 4.6 mm, 5 μm, Dikma Technologies Inc., Beijing, China) with a mobile phase consisting of 0.1 M sodium acetate, 0.1 mM EDTA-2Na, and 10% methanol, adjusted to pH 5.1 with glacial acetic acid. The flow rate was maintained at 1 mL/min. 3,4-dihydroxyphenylacetic acid (DOPAC), a primary intracellular metabolite of dopamine, and homovanillic acid (HVA) were detected at excitation and emission wavelengths of 290 nm and 330 nm, respectively. Both DOPAC and HVA are key dopamine metabolites. Their concentrations relative to DA (DOPAC/DA, HVA/DA) provide a dopamine turnover index, which is widely used to reflect the metabolic activity and functional state of dopaminergic neurons, independent of absolute tissue concentration variations. The concentrations of DA and its metabolites were expressed as nanograms per milligram of tissue weight.

### 2.10. Ribonucleic Acid (RNA) Reverse Transcription and Real-Time Quantitative Polymerase Chain Reaction (qPCR)

Total RNA was isolated from the two cells mentioned earlier using TRIzol reagent (Thermo Fisher Scientific) according to the manufacturer’s instructions. The RNA samples (2 mg) were used for the synthesis of cDNA by reverse transcription using a GoScript Reverse Transcriptase kit (Promega, Madison, WI, USA). Real-time PCR was performed with a CFX96 Touch Real-Time PCR Detection System (Bio-Rad Laboratories, Hercules, CA, USA). The PCR mixture was at a volume of 10 mL containing 5 mL SYBR Premix Ex Taq (Promega), 0.5 mM each of the primers, and 1 mL cDNA prepared as described and using the following conditions: 95 °C for 2 min, 95 °C for 15 s, and 60 °C for 60 s for 40 cycles. The results were analyzed according to the 2-DDCq formula. The sequences of primers are listed in Figure S1.

### 2.11. Rotating Rod Experiment

To evaluate the coordination and balance of mice’s movement, a rotarod test was conducted. All mice were placed on a rotating rod at a constant speed of 5 rpm for 5 min to acclimate. After resting for at least 30 min, the mice were placed back on the rod, which was then accelerated from 5 rpm to 40 rpm over a 10 min period. The latency to fall from the rod was recorded [32].

### 2.12. The Open-Field Experiment

The open-field test is conducted by placing a mouse gently in the center of a large, square, and brightly lit arena, allowing it to freely explore the environment for a standardized period, typically 5 to 10 min, while its movement is digitally recorded by a camera mounted above the apparatus; the subsequent video analysis utilizes tracking software to quantify various behavioral parameters, including total distance traveled, average speed, time spent and distance moved in the center versus the periphery of the arena (to assess anxiety-like behavior based on thigmotaxis, the tendency to stay near walls), and the number of rearings (vertical exploratory activity), with the entire apparatus being thoroughly cleaned with a disinfectant or ethanol solution between each animal trial to remove any olfactory cues that could influence the behavior of subsequent subjects [33].

### 2.13. Pole Climbing Experiment

To assess motor function in mice, a pole climbing test was performed. Each mouse was placed head up at the top of a cylindrical wooden pole (50 cm in length and 2 cm in diameter) with a rough surface. The time taken for the mouse to descend from the top to the base of the pole using its forelimbs was recorded. If a mouse stopped midway or climbed in the wrong direction, the measurement was repeated [33].

### 2.14. Beam Walking Experiment

To test the motor function of mice, a beam walking experiment was conducted. Mice were placed at one end of a square wooden pole (60 cm long and 0.8 cm wide), which was horizontally fixed at a height of 50 cm. The time taken to traverse the crossbar within 60 s was recorded. If a mouse stopped halfway or walked backward, the trial was repeated. Before formal testing, the animals were trained 2–3 times per day for two consecutive days.

### 2.15. RNA-Seq

Differential expression analysis was performed using [e.g., DESeq2]. Genes with an adjusted *p*-value (FDR) < 0.05 and |log2(fold change)| > 1 were considered significantly differentially expressed. Read counts were normalized using the [e.g., median of ratios] method.

### 2.16. Statistical Analysis

Statistical analysis was performed using GraphPad Prism software (version 8.0; GraphPad Software, San Diego, CA, USA). All experimental data are presented as the mean ± standard error of the mean. One-way analysis of variance was used for comparison among multiple groups, whereas Student’s *t*-test was used for comparisons between two groups. Statistical significance was defined as * *p* < 0.05, ** *p* < 0.01, and *** *p* < 0.001.

## 3. Results

### 3.1. BAP31 Is a Potential Regulatory Factor Mediating PD in Mice

**Figure 1:**
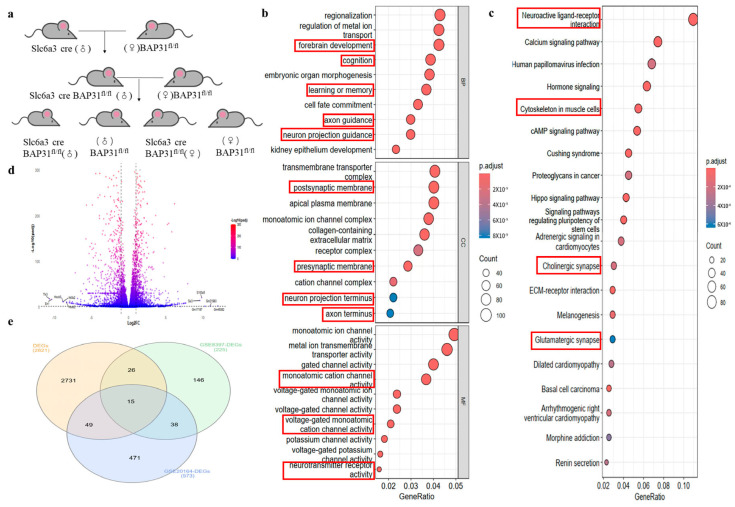
**BAP31 is a potential regulatory factor mediating Parkinson’s disease in mice:** (**a**) Reproductive flowchart of BAP31^fl/fl^ and Slc6a3cre-BAP31^fl/fl^ mice. (**b**) GO annotation classification histogram in the substantia nigra pars compacta of BAP31^fl/fl^ and Slc6a3cre-BAP31^fl/fl^ mice. (**c**) The KEGG enrichment analysis of differentially expressed genes in the substantia nigra pars compacta of BAP31^fl/fl^ and Scl6a3 cre-BAP31^fl/fl^ mice. (**d**) The volcanic pattern analysis of these differentially expressed genes. (**e**) The Venn diagrams of the sample database alongside the two Parkinson’s disease databases, GSE8397-DEGs and GSE20164-DEGs.

To investigate the role of BAP31 in neurons, we created mice with BAP31 condition knocked down. BAP31^fl/fl^ was hybridized with Slc6a3-Cre transgenic mice. The resulting Slc6a3cre-BAP31^fl/fl^ mice were selected as the experimental group, while littermate BAP31^fl/fl^ mice served as the control group. The mouse reproduction flowchart shows the breeding scheme (Figure 1a). To explore functional genomic differences, we performed RNA-seq on substantia nigra tissues from Slc6a3cre-BAP31^fl/fl^ and BAP31^fl/fl^ mice (newly generated data). In parallel, we analyzed public PD-related transcriptomic datasets “GSE8397” and “GSE20164” for comparative and validation purposes. RNA-seq was employed to explore the functional genomic differences between the two groups. To better understand these differences, we performed transcriptome-based gene enrichment analysis on brain tissues from both Slc6a3cre-BAP31^fl/fl^ and BAP31^fl/fl^ mice. In this study, we enriched and analyzed the differentially expressed genes in the substantia nigra pars compacta of BAP31^fl/fl^ and Scl6a3 cre-BAP31^fl/fl^ mice using Gene Ontology (GO) and Kyoto Encyclopedia of Genes and Genomes (KEGG) analyses to investigate their biological functions. The GO analysis revealed that the significantly enriched biological processes (BP) include “learning and memory”, “cognition”, and “forebrain development”. Regarding cellular composition (CC), significant enrichment was observed in presynaptic membranes, postsynaptic membranes, and neuronal projection ends. In terms of molecular function, we identified significant enrichment in “neurotransmitter receptor activity”, “monoatomic cation channel activity”, and “voltage-gated monoatomic cation channel activity” (Figure 1b). The KEGG pathway analysis indicated that the related genes were significantly enriched in “neuroactive ligand–receptor interaction”, “cholinergic synapse”, and “cytoskeleton in muscle cells” (Figure 1c). These findings suggest that the differentially expressed genes may play a crucial role in the onset and metabolism of nervous system diseases. A volcano plot is an effective tool for visualizing the results of gene expression analysis. We found a large number of upregulated and downregulated genes. Among them, the five with the highest degree of upregulation were Six3, S100a5, Gm17167, Gm21983, and Gm45062. Among them, the ones with the highest degree of downregulation are Tlx3, EN1, NrOb2, and Hoxb2 (Figure 1d). We analyzed the PD-related datasets from the database, specifically GSE8397-DEGs and GSE20164-DEGs, and conducted a differential expression analysis. By intersecting these datasets, we identified 15 differentially expressed genes (DEGs) associated with PD (Figure 1e). We also identified some genes in the heatmap results that are associated with Parkinson’s disease (Figure S2).

### 3.2. BAP31 Deficiency Exacerbates Motor Dysfunction in MPTP-Treated Mice

**Figure 2:**
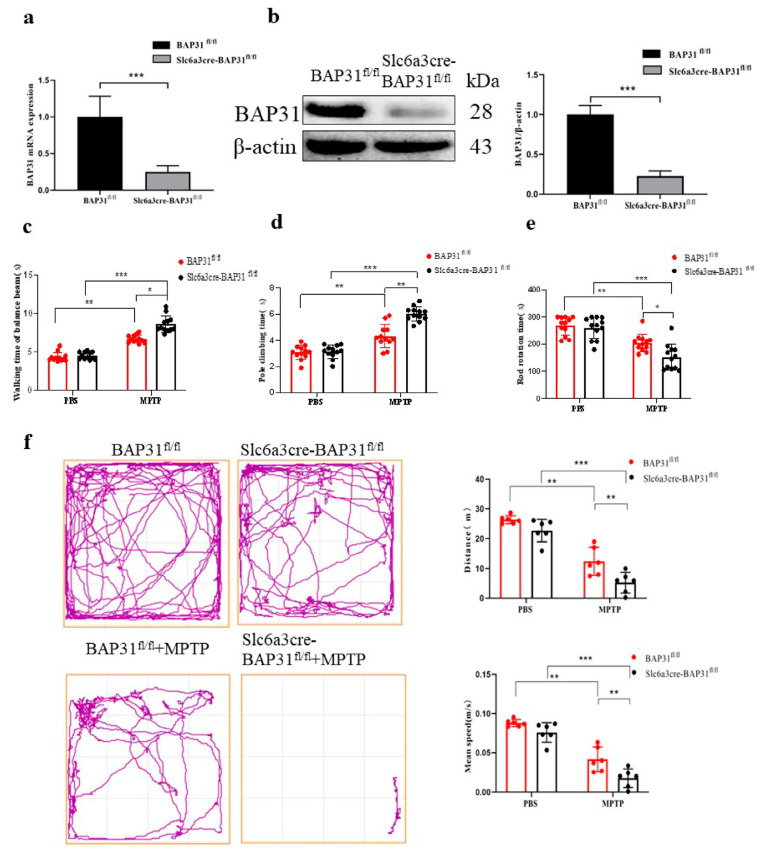
**BAP31 deficiency increased behavioral dysfunction in MPTP-treated mice:** (**a**) mRNA expression of BAP31 in primary DA neurons from Slc6a3cre-BAP31^fl/fl^ and BAP31^fl/fl^ analyzed by real-time PCR (BAP31^fl/fl^ mice, *n* = 3; Slc6a3cre-BAP31^fl/fl^, *n* = 3). (**b**) Protein expression of BAP31 in primary DA neurons from Slc6a3cre-BAP31^fl/fl^ and BAP31^fl/fl^, detected by WB (BAP31^fl/fl^ mice, *n* = 3; Slc6a3cre-BAP31^fl/fl^, *n* = 3). (**c**–**f**) Behavioral results of 6-month-old Slc6a3cre-BAP31^fl/fl^ and BAP31^fl/fl^ mice injected with saline or MPTP (half male and female). (**c**) Walking time of the balance beam test. (**d**) Pole climbing test. (**e**) Rotating rod experiment. (**f**) The open-field test. (Experimental group: BAP31^fl/fl^ mice, *n* = 12; Slc6a3cre-BAP31^fl/fl^ mice, *n* = 12; BAP31^fl/fl^ mice injected with MPTP, *n* = 12; and Slc6a3cre-BAP31^fl/fl^ mice injected with MPTP, *n* = 12). * *p* < 0.05, ** *p* < 0.01, *** *p* < 0.001.

To clarify the role of BAP31 expression in the pathogenesis of PD, we evaluated whether changes in BAP31 levels would influence PD progression in mice. 1-methyl-4-phenyl-1,2,3,6-tetrahydropyridine (MPTP) is a neurotoxin commonly used to establish animal models of PD in laboratory settings. It selectively damages dopaminergic neurons, leading to motor dysfunction symptoms that closely resemble the clinical features of human PD. First, we examined the protein and mRNA levels of BAP31 in primary DA neurons from Slc6a3cre-BAP31^fl/fl^ and BAP31^fl/fl^. The results showed that the expression of BAP31 was significantly reduced in primary DA neurons from Slc6a3cre-BAP31^fl/fl^ mice (Figure 2a,b). We determined that BAP31^fl/fl^ mice were not significantly different from Slc6a3-Cre mice in their behavior (Figure S3). To evaluate PD-related behaviors, we conducted a series of tests, including the rotarod test, pole-climbing test, beam walking test, and the open-field test, to evaluate motor coordination and muscle strength. No baseline behavioral differences were observed between Slc6a3cre-BAP31^fl/fl^ and BAP31^fl/fl^ mice. However, following MPTP injection, Slc6a3cre-BAP31^fl/fl^ mice exhibited a significantly shorter walking time in the crossbar test (Figure 2c). In the climbing pole experiment, after MPTP injection, the climbing pole time of BAP31^fl/fl^ mice was significantly prolonged compared with that of slc6a3cre-BAP31^fl/fl^ mice (Figure 2d). BAP31 deficiency exacerbates MPTP-lesioned motor coordination injury, as evidenced by a reduction in rotarod performance time (Figure 2e). In the open-field test, MPTP-lesioned Slc6a3cre-BAP31^fl/fl^ mice also exhibited a shorter distance and mean speed than their MPTP-treated BAP31^fl/fl^ counterparts (Figure 2f). These data demonstrate that BAP31 deficiency exacerbates motor coordination deficits in MPTP-treated mice.

### 3.3. BAP31 Deficiency Exacerbates Neuronal Loss in MPTP-Treated Mice

**Figure 3:**
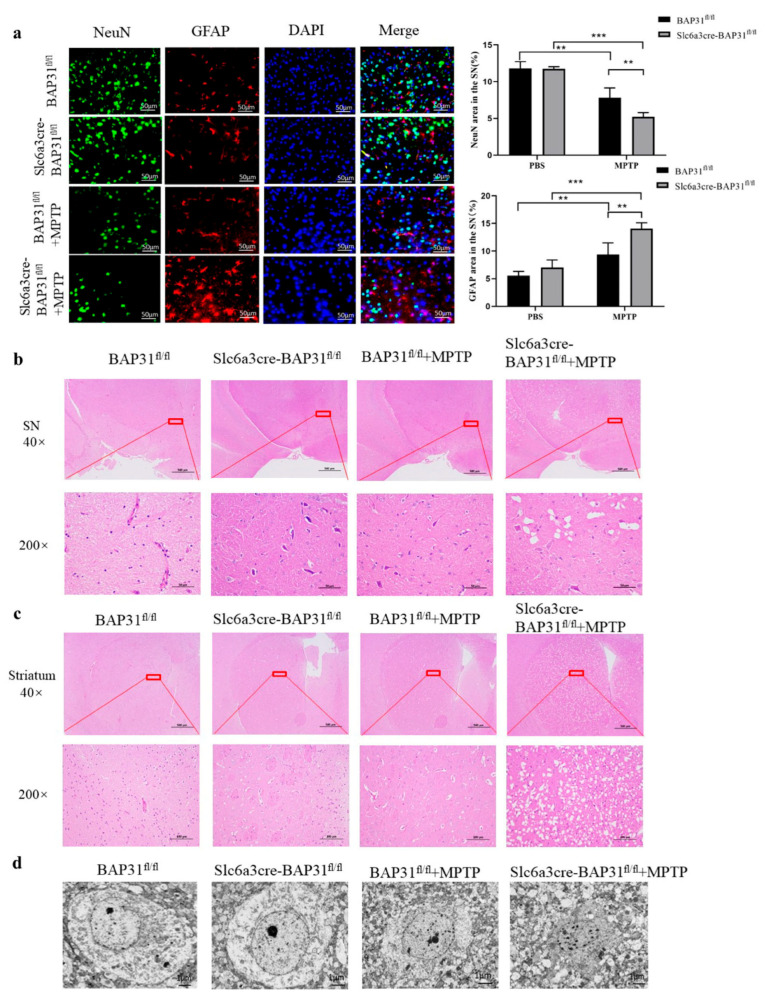
**BAP31 deficiency increased neuron loss induced by MPTP-treated mice:** (**a**) Expression levels of NeuN and GFAP were detected by immunofluorescence assay. Scale bar = 50 μm. Experimental groups: BAP31^fl/fl^ mice, *n* = 3; Slc6a3cre-BAP31^fl/fl^ mice, *n* = 3; BAP31^fl/fl^ mice injected with MPTP, *n* = 3; and Slc6a3cre-BAP31^fl/fl^ mice injected with MPTP, *n* = 3. (**b**) Results of HE staining in the midbrain of mice. Scale bar = 500 or 100 μm. (**c**) Results of HE staining in the striatum of mice. Scale bar = 500 or 100 μm. Experimental groups: BAP31^fl/fl^ mice, *n* = 3; Slc6a3cre-BAP31^fl/fl^ mice, *n* = 3; BAP31^fl/fl^ mice injected with MPTP, *n* = 3; and Slc6a3cre-BAP31^fl/fl^ mice injected with MPTP, *n* = 3. (**d**) The ultrastructure of the nucleus in the midbrain and striatum of mice was observed by an electron microscope. Scale bar  =  1 μm. Experimental groups: BAP31^fl/fl^ mice, *n* = 3; Slc6a3cre-BAP31^fl/fl^ mice, *n* = 3; BAP31^fl/fl^ mice injected with MPTP, *n* = 3; and Slc6a3cre-BAP31^fl/fl^ mice injected with MPTP *n* = 3. ** *p* < 0.01, *** *p* < 0.001.

To systematically investigate the neuroprotective role of BAP31 in PD pathogenesis, we performed dual biomarker analysis using NeuN (a neuronal nuclear marker) and GFAP (an astrocytic marker). The inverse correlation between GFAP upregulation (indicative of reactive gliosis) and NeuN downregulation (reflective of neuronal loss) provides complementary insights into neuronal loss. Immunofluorescence analysis revealed a marked reduction in NeuN protein levels coupled with an increase in GFAP cerebral cortex lysates of MPTP-lesioned Slc6a3cre-BAP31^fl/fl^ mice compared to BAP31^fl/fl^ controls (Figure 3a). At the same time, the protein levels of SYP and PSD95 also decreased significantly (Figure S4). Hematoxylin–eosin (HE) staining was performed on the substantia nigra and striatum of mice. In the control group, the structure of neurons in the substantia nigra and striatum was intact, the nucleus was visible, and no obvious abnormality was observed. Compared with BAP31^fl/fl^ mice, MPTP-lesioned slc6a3cre-BAP31^fl/fl^ mice showed abnormal morphology of some cells in the substantia nigra and striatum, widened intercellular space, hyperchromatic and shrunken nuclei in the substantia nigra, and reduced cell number in the striatum (Figure 3b,c).

Ultrastructural analysis through transmission electron microscopy further delineated the degenerative changes. BAP31-deficient neurons in MPTP-lesioned mice exhibited nuclear envelope irregularities, including invaginations and focal discontinuities, chromatin margination with electron-dense aggregates, perinuclear accumulation of lipofuscin granules, and disrupted nucleoplasmic–cytoplasmic boundary integrity. These multilevel observations collectively demonstrate that BAP31 deficiency exacerbates neuronal loss in MPTP-treated mice.

### 3.4. BAP31 Deficiency Exacerbates Dopaminergic Neurodegeneration in the Substantia Nigra Pars Compacta of MPTP-Treated Mice

**Figure 4:**
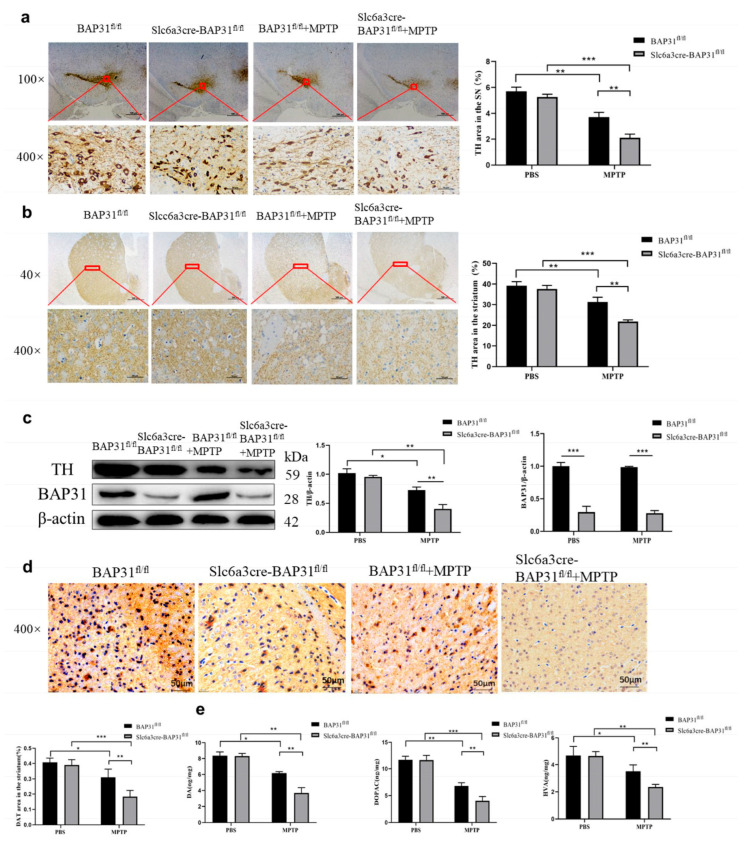
**BAP31 deficiency increased dopamine neuron loss in MPTP-treated mice:** (**a**,**b**) TH content in the midbrain and striatum of mice was detected by immunohistochemistry. Scale bar = 500, 200, or 50 μm. (**c**) TH level in the midbrain and striatum of mice was detected by WB. (**d**) BAP31 deficiency affected the DAT level in the brain of MPTP-treated mice. Scale bar = 50 μm. (**e**) ELISA detected the DA, DOPAC, and HVA levels in the midbrain and striatum of the brains of mice. Experimental groups: BAP31^fl/fl^ mice, *n* = 3; Slc6a3cre-BAP31^fl/fl^ mice, *n* = 3; BAP31^fl/fl^ mice injected with MPTP, *n* = 3; and Slc6a3cre-BAP31^fl/fl^ mice injected with MPTP *n* = 3. * *p* < 0.05, ** *p* < 0.01, *** *p* < 0.001.

PD is a neurodegenerative disorder characterized by the gradual loss of dopaminergic neurons in the substantia nigra of the midbrain, leading to decreased dopamine levels and resulting in dyskinesia and other related symptoms. TH is a key enzyme for synthesizing dopamine; it converts tyrosine into dopa, the first step in dopamine synthesis [34]. We found that, compared with MPTP-treated BAP31^fl/fl^ mice, BAP31 deletion can aggravate MPTP-lesioned loss of TH^+^ neurons in the striatum of PD mice and decreases TH levels in the striatum (Figure 4a). Additionally, we found the same pattern in the midbrain (Figure 4b). TH plays a vital role in dopamine synthesis. The results of WB indicated that BAP31 deletion exacerbates the MPTP-lesioned reduction in TH levels in the striatum of PD mice compared with BAP31^fl/fl^ mice (Figure 4c). The dopamine transporter (DAT) is a crucial marker of presynaptic dopaminergic terminals. DAT is located on the presynaptic membrane of dopamine neurons [35]. Its function is to transport dopamine released into the synaptic space back to the presynaptic neuron through active transport, thereby maintaining normal synaptic physiological function. DAT can be used to evaluate the functional state of dopaminergic nerve fiber endings in the striatum. We detected the expression level of DAT in the striatum, and the results showed that BAP31 deletion could exacerbate the significant reduction in DAT levels in the striatum of PD mice injected with MPTP compared with BAP31^fl/fl^ mice (Figure 4d). We then used an ELISA kit to measure DA, DOPAC, and HVA content in the midbrain and striatum. The results indicated that BAP31 deletion significantly worsened the MPTP-lesioned decrease in DA and DOPAC content in both regions compared with BAP31^fl/fl^ mice. Compared with BAP31^fl/fl^ mice, BAP31 deletion significantly exacerbated the increase in HVA content in both regions caused by the MPTP-treated decrease (Figure 4e).

### 3.5. BAP31 Deficiency Exacerbates Mitochondrial Imbalance in MPTP-Treated Mice

**Figure 5:**
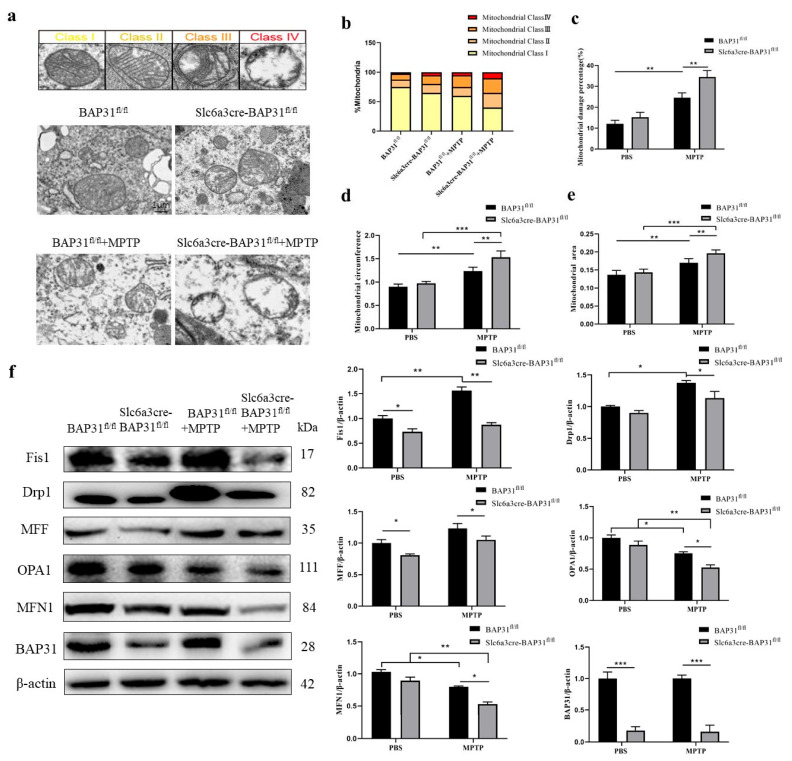
**BAP31 deficiency results in MPTP-lesioned mitochondrial homeostasis in PD mice:** (**a**–**e**) The ultrastructure of mitochondria in the midbrain and striatum of mice was observed using electron microscopy. Mitochondrial status was rated as I–IV, and the number of different grades was counted. Scale bar  =  1 μm. (**f**) The content of BAP31, MFF, Drp1, FIS1, MFN1, and OPA1 in the midbrain and striatum was detected by WB. Experimental groups: BAP31^fl/fl^ mice, *n* = 3; Slc6a3cre-BAP31^fl/fl^ mice, *n* = 3; BAP31^fl/fl^ mice injected with MPTP, *n* = 3; and Slc6a3cre-BAP31^fl/fl^ mice injected with MPTP, *n* = 3. * *p* < 0.05, ** *p* < 0.01, *** *p* < 0.001.

Mitochondrial homeostasis is closely related to PD. Maintaining mitochondrial homeostasis helps to ensure the energy supply of cells, thus improving the survival rate of dopaminergic neurons. Mitochondria are organelles involved in energy metabolism, and they also participate in cell differentiation and apoptosis, as well as regulate cell growth and cell cycle [36]. Under pathological conditions, the shape, structure, and function of mitochondria can change in volume, quantity, and shape due to changes in the internal and external cellular environment. According to Vaillant–Beuchot’s report, mitochondria can be divided into the following four categories according to their morphology. Type I: mitochondria have high electron density, uniform matrix, and dense ridges with regular distribution; Type II: mitochondrial cristae are slightly broken, and the electron density of the matrix decreases; Type III: mitochondrial cristae are extensively broken, and vacuoles appear in mitochondria; and Type IV: the mitochondrial membrane breaks and swells, becoming transparent and vacuolated [4,37,38]. The results of transmission electron microscopy revealed that BAP31 deletion exacerbates mitochondrial damage in the striatum of PD mice lesioned by MPTP, compared to BAP31^fl/fl^ mice treated with MPTP (Figure 5a–e). The dynamic changes in mitochondria, especially the processes of fission and fusion, are crucial for maintaining mitochondrial function and cellular health [39,40]. These processes are regulated by a specific set of proteins, including the following: Mitochondrial fission proteins include Dynamin-related protein 1 (Drp1), a key mitochondrial division protein that forms a ring structure in the inner membrane and promotes mitochondrial division, and mitochondrial fission 1 protein (Fis1), which plays an auxiliary role in recruiting Drp1 to the mitochondrial membrane, promoting mitochondrial division. Mitochondrial fusion proteins include MFN1 and MFN2 (Mitofusins 1 and 2). These two proteins are responsible for the fusion of the mitochondrial outer membrane. They can form homologous or heterodimers that promote the fusion of the two mitochondria. Optic atrophy 1 (OPA1): OPA1 plays a role in fusion on the inner membrane and also participates in the structure maintenance of the mitochondrial inner membrane and the regulation of mitochondrial function. Through the WB experiment, we found that BAP31 deficiency led to a significant decrease in the protein level of mitochondrial division and fusion protein in MPTP-treated mice (Figure 5f), resulting in an imbalance in mitochondrial homeostasis.

### 3.6. BAP31 Deficiency Affects the Expression of PINK1/Parkin Pathway-Related Proteins in MPTP-Treated Mice

**Figure 6:**
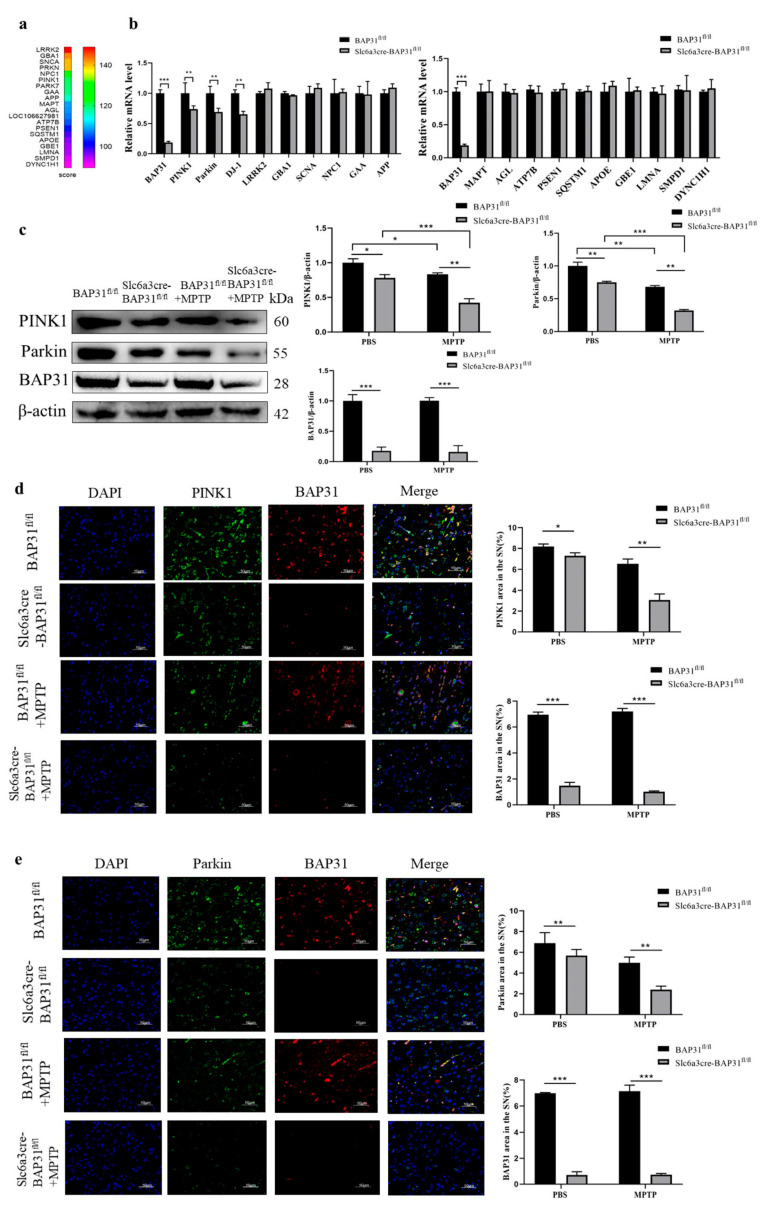
**BAP31 deficiency affected the expression of PINK1/Parkin pathway-related proteins in MPTP-treated mice:** (**a**) GeneCard scores of PD-related genes, indicating 19 genes with scores above 90. (**b**) The mRNA levels of each protein in the mouse brain were analyzed by qPCR. (**c**) The protein expressions of BAP31, PINK1, and Parkin were detected and quantitatively analyzed by WB. (**d**,**e**) The results of immunofluorescence double staining indicated the fluorescence levels of PINK1 and Parkin in the midbrain and striatum of mice. Scale bar = 50 μm. Experimental groups: BAP31^fl/fl^ mice, *n* = 3; Slc6a3cre-BAP31^fl/fl^ mice, *n* = 3; BAP31^fl/fl^ mice injected with MPTP, *n* = 3; and Slc6a3cre-BAP31^fl/fl^ mice injected with MPTP, *n* = 3. * *p* < 0.05, ** *p* < 0.01, *** *p* < 0.001.

The PINK1/Parkin pathway is crucial for maintaining mitochondrial homeostasis and neuronal health, and its dysfunction is closely related to the occurrence and progression of PD. Currently, the pathogenic genes of PD remain unclear. We selected 19 genes with a score above 90 points, related to PD, from the gene pool; the higher the correlation, the higher the score (Figure 6a). We also performed a similar experiment in SH-SY5Y cells, where BAP31 was knocked down, and the mRNA levels of PD-related genes were detected; the results were the same as those in animal experiments (Figure S5). Subsequently, mRNA levels of 19 genes in BAP31^fl/fl^ mice and Slc6a3cre-BAP31^fl/fl^ were detected by q-PCR. The results indicated that mRNA levels of three genes-*PINK1*, *Parkin*, and *DJ-1* changed significantly (Figure 6b). While DJ-1 mRNA was also significantly reduced, subsequent protein-level validation focused on PINK1 and Parkin due to their direct role in the mitochondrial autophagy pathway, central to our mechanistic hypothesis. Subsequently, WB was used to detect the expression levels of BAP31, PINK1, and Parkin proteins, and it was found that BAP31 deficiency significantly reduced PINK1 and Parkin protein levels in the midbrain and striatum of MPTP-treated mice (Figure 6c). Later, we further verified through immunofluorescence that BAP31 deficiency led to a decrease in PINK1 and Parkin levels in the striatum of MPTP-treated mice (Figure 6d,e).

### 3.7. BAP31 Regulates the Levels of PINK1 via EN1

**Figure 7:**
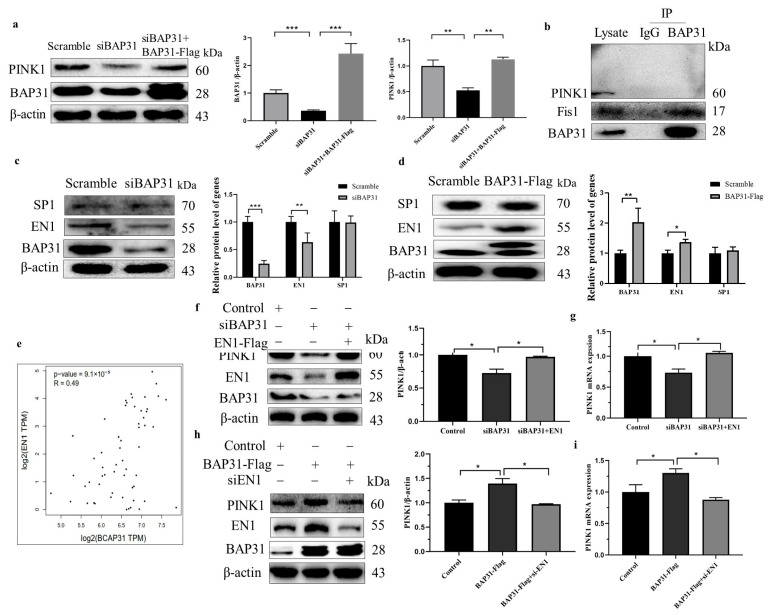
**BAP31 regulated the levels of PINK1 through EN1:** (**a**) SH-SY5Y was transfected with siBAP31, and a Flag tag and WB were performed to observe changes in PINK1 protein levels. (**b**) Co-IP results indicate BAP31 and PINK1. (**c**) SH-SY5Y cells were transfected with siBAP31, and changes in SP1 and EN1 protein levels were observed by WB. (**d**) SH-SY5Y cells were transfected with Flag, and changes in SP1 and EN1 protein levels were detected by WB. (**e**) Correlation analysis of BAP31 and EN1. (**f**) WB analysis of PINK1 expression in cells transfected with siRNA-BAP31 and overexpressing EN1. (**g**) Analysis of the mRNA level of PINK1 expression in cells transfected with siRNA-BAP31 and those overexpressing EN1. (**h**) WB analysis of PINK1 expression in cells transfected with siRNA-EN1 and overexpressing BAP31. (**i**) Analysis of the mRNA level of PINK1 expression in cells transfected with siRNA-EN1 and overexpressing BAP31. * *p* < 0.05, ** *p* < 0.01, *** *p* < 0.001.

To further verify the results, we used WB to measure the expression of PINK1 in SH-SY5Y cells transfected with siRNA-BAP31. The results indicated that PINK1 expression decreased after BAP31 knockdown in SH-SY5Y cells. We then overexpressed BAP31-Flag in siRNA-BAP31-transfected cells, and WB analysis revealed that overexpressed BAP31 reversed the decrease in PINK1 expression levels (Figure 7a). We speculated that BAP31 might interact with PINK1. Subsequently, immunoprecipitation analysis using a co-immunoprecipitation (Co-IP) experiment revealed no binding relationship between BAP31 and PINK1 in SH-SY5Y cells (Figure 7b). By screening three PD-related databases, we found that SP1 and EN1 were promoters of PINK1. We then measured the expression of EN1 and SP1 in SH-SY5Y cells of siRNA-BAP31. The results exhibited that the protein expression level of SP1 did not change significantly, but EN1 expression was significantly decreased (Figure 7c). Subsequently, we overexpressed BAP31-Flag, and the results demonstrated that the protein expression level of SP1 did not change significantly, but EN1 expression was significantly increased (Figure 7d). The results of the correlation analysis indicated a significant relationship between BAP31 and EN1. (Figure 7e). In BAP31 knockdown cells with EN1 overexpression, WB analysis revealed a significant increase in PINK1 levels compared to BAP31 knockdown cells without EN1 overexpression (Figure 7f). Additionally, the mRNA levels of PINK1 were also significantly elevated (Figure 7g). Conversely, in EN1-knockdown cells that overexpress BAP31, WB analysis indicated a marked decrease in PINK1 levels compared to BAP31-overexpressing cells (Figure 7h). Furthermore, the mRNA levels of PINK1 were significantly reduced following EN1 knockdown (Figure 7i). These findings suggest that BAP31 regulates PINK1 mRNA levels through EN1.

### 3.8. BAP31 Depletion Affects PINK1–Parkin Pathway and Mitochondrial Homeostasis Through Decreasing EN1 Expression in PD

**Figure 8:**
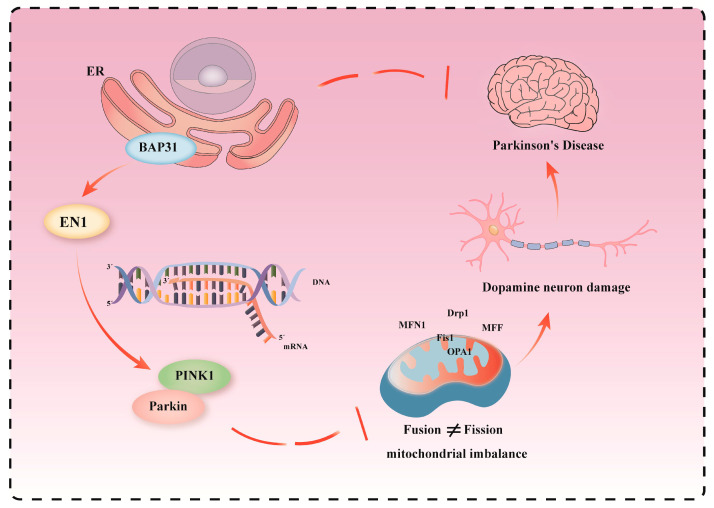
We used arrowheads (→) to indicate activation or positive regulation and T-shaped arrowheads (--|) to indicate inhibition or negative regulation. In this study, we found that BAP31 deficiency down-regulated the PINK1 transcription factor EN1, thereby impairing the PINK1–Parkin pathway. This disruption led to an imbalance in mitochondrial fission and fusion, mitochondrial dysfunction, loss of dopamine neurons, and ultimately exacerbated PD development.

The results of our study are summarized in a schematic diagram (Figure 8). Collectively, our findings demonstrate that BAP31 regulates mitochondrial homeostasis through the PINK1–Parkin pathway in PD. Specifically, we showed that BAP31 deficiency downregulates the PINK1 transcription factor EN1, thereby impairing the PINK1–Parkin pathway. This disruption leads to an imbalance in mitochondrial fission and fusion, resulting in mitochondrial dysfunction, loss of dopamine neurons, and ultimately accelerated PD development.

## 4. Discussion

BAP31, an endoplasmic reticulum (ER)-resident transmembrane protein, was originally characterized through its preferential interaction with IgD—a key antigen receptor on mature B lymphocytes, leading to its designation as B-cell receptor-associated protein 31 [12,41,42]. Functionally, BAP31 serves as a multifunctional chaperone that regulates both the ER-associated degradation (ERAD) of misfolded polypeptides and caspase-8-mediated apoptotic signaling cascades, while also coordinating the retrograde transport of membrane proteins [43,44]. Emerging evidence implicates BAP31 in neurodevelopment and neurodegeneration. Immunohistochemical analyses reveal enriched BAP31 expression in cortical and nigral neurons, where it modulates activity-dependent synaptic pruning through ER–mitochondria crosstalk [45,46]. Clinically, loss-of-function mutations in BAP31 are associated with motor neuropathies and cerebellar ataxia in humans, characterized by axonal dieback and Lewy body-like inclusions. While preliminary studies suggest BAP31 dysregulation in AD pathogenesis, particularly in amyloid precursor protein (APP) trafficking, its role in PD remains unexplored [47,48]. Notably, PD and AD share convergent mechanisms, including ER stress, proteostasis failure, and mitochondrial dysfunction. Given BAP31’s established roles in ER quality control and apoptotic regulation, we hypothesized that BAP31 deficiency may exacerbate α-synucleinopathy-associated neurodegeneration.

To test this, we generated dopaminergic neuron-specific BAP31 cKO mice-Slc6a3cre BAP31^fl/fl^, and subjected them to subacute MPTP-probenecid challenge; key findings are related to motor phenotype, namely deterioration of motor coordination and locomotor performance. The MPTP model represents toxin-induced Parkinsonism rather than idiopathic PD. As evidenced by four independent behavioral metrics: in the open-field test, MPTP-lesioned Slc6a3cre-BAP31^fl/fl^ mice also exhibited a shorter distance and mean speed than their MPTP-treated BAP31^fl/fl^ counterparts, significantly reduced latency to fall in the accelerating rotarod test, prolonged traversal duration during the balance beam walking assay, and substantially elevated time-to-descend in the pole climbing test, collectively indicating profound motor deficits across multiple neurological domains. However, BAP31 conditional deletion shows no baseline phenotype, with deficits emerging only after MPTP. While our behavioral assessment demonstrated consistent motor deficits in BAP31-deficient mice after MPTP challenge, it primarily relied on global measures of coordination, endurance, and locomotion (e.g., rotarod latency, beam traversal time). Future studies could benefit from incorporating more dopamine-sensitive, fine-motor tasks, such as quantification of beam slips or foot faults, which have been shown to robustly correlate with nigrostriatal degeneration in transgenic PD models [49]. The genetic background and subline differences (such as C57BL/6J and C57BL/6N) have a profound impact on neurochemical and behavioral phenotypes [50,51]. A limitation of this study is its use of a single genetic background (C57BL/6J). Although this provided an advantage for internal mechanistic comparisons, it reduces the generalizability of the absolute phenotypic results. Therefore, future work is needed to validate the findings in other genetic backgrounds or Parkinson’s disease models. Such measures may reveal even earlier or more specific aspects of motor impairment linked to BAP31 dysfunction. While our study included both sexes and found no evidence that sex modulated the primary effects of BAP31 deficiency on MPTP-induced deficits, future work in larger cohorts could more precisely determine whether sex influences the susceptibility or progression of pathology in this pathway. An important consideration for the interpretation of neurochemical and behavioral studies is the influence of endogenous seasonal rhythms [52], which can modulate monoaminergic and neuroendocrine systems independently of immediate laboratory housing conditions. While the core comparative findings of this study are internally robust—as all experimental and control groups were processed within the same seasonal window—the absolute values may be season-dependent. Future replication across multiple seasons would strengthen the generalizability of the results.

Neuropathology: stereological counts revealed a reduction in tyrosine hydroxylase-positive (TH^+^) neurons in the substantia nigra pars compacta (SNpc) of Slc6a3cre BAP31^fl/fl^ +MPTP mice versus in BAP31^fl/fl^ +MPTP controls; Dopaminergic Markers: IF and WB quantification demonstrated reductions in striatal TH and DAT protein levels in Slc6a3cre BAP31^fl/fl^ +MPTP mice. Specifically, the observation that BAP31-deficient mice without MPTP exposure showed histological alterations (e.g., striatal cell loss in Figure 3c) reminiscent of WT mice with MPTP suggests that BAP31 loss may establish a pro-degenerative cellular state or increase basal neuronal vulnerability. These changes likely represent early-stage vulnerability markers (e.g., dysregulated proteostasis, subthreshold neuroinflammation) rather than overt, rapid neurodegeneration, as significant motor deficits in our model were only unmasked following an additional metabolic or toxic stressor (MPTP). These results establish BAP31 as a novel neuroprotective factor in PD pathogenesis, potentially through dual regulation of ER–mitochondrial proteostasis and dopaminergic circuit integrity.

Mitochondrial dysfunction and impaired fission–fusion equilibrium are central to PD pathogenesis. As cellular powerhouses, mitochondria govern ATP synthesis, calcium homeostasis, and apoptotic regulation—processes particularly critical for energy-demanding dopaminergic neurons [53,54]. Our study demonstrates that BAP31 knockdown exacerbates mitochondrial ultrastructural damage in MPTP-intoxicated mice. The balance between mitochondrial fission (mediated by Drp1) and fusion (regulated by MFN1/2 and OPA1) is essential for maintaining functional networks [55,56]. Quantitative immunoblotting revealed that BAP31 deficiency disrupts this equilibrium, reducing both fission and fusion protein levels. qPCR analysis identified 19 pathogenic genes most strongly associated with PD, among which only the expression levels of PINK1 and Parkin were significantly decreased. Since PINK1 and Parkin are closely related to mitochondria, we further confirmed through WB analysis that the expression levels of PINK1 and Parkin in the striatum and midbrain of mice were significantly decreased. Additionally, immunofluorescence analysis revealed that BAP31 knockdown led to decreased levels of PINK1 and Parkin in the midbrain of MPTP-induced PD mice. Given that BAP31 modulates the mRNA expression of PINK1, suggesting a pre-transcriptional regulatory mechanism, we first investigated whether BAP31 directly interacts with PINK1. However, Co-IP assays revealed no direct physical interaction between BAP31 and PINK1. This observation prompted us to hypothesize that BAP31 may instead regulate PINK1 expression by influencing its upstream transcriptional activators.

Our findings position BAP31 as an upstream modulator of this pathway. Initial screening of PINK1 promoter databases (JASPAR, TRANSFAC) prioritized EN1 and SP1 as candidate regulators. In SH-SY5Y cells, BAP31 knockdown selectively reduced EN1 protein levels, while SP1 remained unchanged. Functional rescue experiments demonstrated EN1’s pivotal role: transient overexpression in BAP31-KO cells restored PINK1 to baseline levels. Based on these findings, we propose that BAP31 regulates the PINK1–Parkin signaling pathway via the transcription factor EN1, thereby contributing to the amelioration of behavioral deficits and pathological alterations in PD mouse models. This mechanistic cascade underscores BAP31’s potential role in modulating mitochondrial quality control and neuronal survival through EN1-dependent transcriptional regulation. The PINK1–Parkin pathway, central to mitophagy and mitochondrial quality control, functionally intersects with EN1-mediated mitochondrial homeostasis [57], as EN1 modulates oxidative phosphorylation genes that influence PINK1-dependent Parkin recruitment and damaged organelle clearance. EN1 orchestrates PINK1 transcription via direct interactions with its promoter region, creating a neuroprotective axis against oxidative stress [24,58,59]. This regulatory mechanism operates through three interdependent pathways: (1) enhancing PINK1-driven mitophagy, (2) stabilizing mitochondrial membrane potential, and (3) suppressing ROS accumulation. Mechanistically, as an ER membrane protein, BAP31 might facilitate EN1 protein folding, trafficking, or stability via ER quality control systems, ensuring its functional availability in neuronal maintenance. Further studies are warranted to elucidate the underlying molecular mechanisms. This mechanistic triad—BAP31/EN1/PINK1—provides a unified framework linking ER proteostasis, transcriptional regulation, and mitochondrial resilience in PD pathogenesis. These findings establish BAP31 as a master coordinator of dopaminergic survival through EN1-PINK1 orchestration, offering therapeutic targets to enhance mitochondrial resilience in neurodegenerative proteinopathies. While our mechanistic investigation focused on the BAP31–EN1–PINK1 axis, Parkinson’s disease is characterized by a complex interplay of multiple genetic networks affecting neuronal survival, mitochondrial function, and synaptic integrity [60]. The transcriptional changes observed in BAP31-deficient mice—including alterations in genes involved in neurodevelopment and synaptic signaling (Figure 1b,c)—resonate with the view that PD-associated genes often converge on common functional modules. Future studies that systematically map interactions between BAP31 and other PD-related gene products, particularly those involved in endoplasmic reticulum–mitochondria crosstalk and protein quality control, will further clarify its position within the broader PD pathogenic network.

In conclusion, our study elucidates BAP31 as a critical neuroprotective regulator in PD pathogenesis, integrating endoplasmic reticulum proteostasis with mitochondrial resilience through the EN1-PINK1 axis. BAP31 deficiency exacerbates α-synucleinopathy-associated neurodegeneration by disrupting mitochondrial fission–fusion dynamics, impairing PINK1–Parkin-mediated, amplifying dopaminergic neuron loss. Mechanistically, BAP31 governs the transcription factor EN1, which directly activates PINK1 expression, thereby stabilizing mitochondrial quality control. Our findings highlight BAP31 as a master coordinator of dopaminergic circuit integrity and a potential therapeutic target to enhance mitochondrial resilience in neurodegenerative disorders, offering new strategies to counteract proteostasis failure in PD and related proteinopathies.

## 5. Conclusions

In conclusion, we found that the deficiency of BAP31 disrupts mitochondrial homeostasis by reducing the expression of the transcription factor EN1 and inhibiting the expression of PINK1, aggravating motor dysfunction and substantia nigrostriatal degeneration. It is accompanied by a decrease in tyrosine hydroxylase (TH) levels and an increase in neuronal susceptibility, resulting in a reduction in the number of dopamine neurons and insufficient neuronal energy, thereby exacerbating the pathology of PD. The current study identified BAP31 as a critical upstream regulator of PINK1-mediated mitochondrial quality control, highlighting its therapeutic potential for neuroprotection in PD.

## Data Availability

All data generated or analyzed during this study are included in this published article and its Supplementary Information Files.

## Supplementary Materials

The following supporting information can be downloaded at: https://www.mdpi.com/article/10.3390/cells15020137/s1, Figure S1: The sequence encoding of mRNA. Figure S2: Shows the heat map analysis of differentially expressed genes in the brain of BAP31^fl/fl^ and Scl6a3 cre-BAP31^fl/fl^ mice. Figure S3: Behavioral results (Rotating rod experiment, Pole climbing experimentand Beam walking experiment) of 6-month-old Slc6a3-Cre and BAP31^fl/fl^ mice injected with PBSorMPTP. (Experimental groups: BAP31^fl/fl^ mice, n = 6; Slc6a3cre-BAP31^fl/fl^ mice, n = 6; BAP31^fl/fl^ mice injected with MPTP, n = 6; and Slc6a3cre-BAP31^fl/fl^ mice injected with MPTP, n = 6). Figure S4: The content of SYP, PSD95 in the mid brain and striatum were detected by WB. (Experimental groups: BAP31^fl/fl^ mice, n = 3; Slc6a3cre-BAP31^fl/fl^ mice, n = 3; BAP31^fl/fl^ mice injected with MPTP, n = 3; and Slc6a3cre-BAP31^fl/fl^ mice injected with MPTP, n = 3). Figure S5: The mRNA levels of each protein in the SH-SY5Y cells were analyzed by qPCR.

## Abbreviations

PD	Parkinson’s disease
MPTP	1-methyl-4-phenyl-1,2,3,6-tetrahydropyridine
AD	Alzheimer’s disease
EN1	Engrailed Homeobox 1
DA	Determination of dopamine
TH	Hydroxylase
PINK1	PTEN-induced kinase 1
ER	Endoplasmic reticulum
DAT	Dopamine transporter
ROS	Reactive oxygen species
Drp1	Dynamin-related protein 1
Fis1	Fission 1 protein
OPA1	Optic atrophy 1
APP	Amyloid precursor protein

## References

[1] Chiaro G., Vichayanrat E., Koay S., Rogeau A., Ingle G.T., McNamara P., Watson L., Bomanji J., Mathias C.J., Iodice V. (2025). Pure Autonomic Failure: A Natural History Study of the Queen Square Cohort. Brain.

[2] Zhu B., Feng J., Liang X., Fu Z., Liao M., Deng T., Wang K., Xie J., Chi J., Yang L. (2025). Trem2 Deficiency Exacerbates Cognitive Impairment by Aggravating Alpha-Synuclein-Induced Lysosomal Dysfunction in Parkinson’s Disease. Cell Death Discov..

[3] Lind-Holm Mogensen F., Seibler P., Grunewald A., Michelucci A. (2025). Microglial Dynamics and Neuroinflammation in Prodromal and Early Parkinson’s Disease. J. Neuroinflamm..

[4] Bradley J.M., Bugg Z., Pullin J., Moore G.R., Svistunenko D.A., Le Brun N.E. (2025). Human Mitochondrial Ferritin Exhibits Highly Unusual Iron-O(2) Chemistry Distinct from that of Cytosolic Ferritins. Nat. Commun..

[5] Leventhal M.J., Zanella C.A., Kang B., Peng J., Gritsch D., Liao Z., Bukhari H., Wang T., Pao P.C., Danquah S. (2025). An Integrative Systems-Biology Approach Defines Mechanisms of Alzheimer’s Disease Neurodegeneration. Nat. Commun..

[6] Ashton N.J., Benedet A.L., Molfetta G.D., Pola I., Anastasi F., Fernandez-Lebrero A., Puig-Pijoan A., Keshavan A., Schott J., Tan K. (2025). Biomarker Discovery in Alzheimer’s and Neurodegenerative Diseases Using Nucleic Acid Linked Immuno-Sandwich Assay. Alzheimers Dement..

[7] Wyman-Chick K.A., Ferman T.J., Armstrong M.J., Chrenka E.A.B., Chiu S.Y., Patel B., Barrett M.J., Bayram E. (2025). Sex Differences in Prodromal Dementia with Lewy Bodies Using the National Alzheimer’s Coordinating Center data. Alzheimers Dement..

[8] Liu L., Weng Q., Cai Q., Yu X., Huang W., Xie S., Shi Y., Li H., Zhang Y., Hu J. (2025). Choroid Plexus Enlargement Contributes to Motor Severity Via Regional Glymphatic Dysfunction in Parkinson’s Disease. NPJ Park. Dis..

[9] Kallunki P., Sotty F., Willen K., Lubas M., David L., Ambjorn M., Bergstrom A.L., Buur L., Malik I., Nyegaard S. (2025). Rational Selection of the Monoclonal Alpha-Synuclein Antibody Amlenetug (Lu Af82422) for the Treatment of Alpha-Synucleinopathies. NPJ Park. Dis..

[10] Buck S.A., Mabry S.J., Kunkhyen T., Yang Z., Rubin S.A., Yang J., Cheetham C.E.J., Freyberg Z. (2025). Dvglut Is a Mediator of Sex Differences in Dopamine Neuron Mitochondrial Function Across Aging and in a Parkinson’s Disease Model. Aging Cell.

[11] Recinto S.J., Kazanova A., Liu L., Cordeiro B., Premachandran S., Bessaiah H., Allot A., Afanasiev E., Mukherjee S., Pei J. (2025). PINK1 Deficiency Rewires Early Immune Responses in a Mouse Model of Parkinson’s Disease Triggered by Intestinal Infection. NPJ Park. Dis..

[12] Adachi T., Schamel W.W., Kim K.M., Watanabe T., Becker B., Nielsen P.J., Reth M. (1996). The Specificity of Association of the Igd Molecule with the Accessory Proteins Bap31/Bap29 Lies in the Igd Transmembrane Sequence. EMBO J..

[13] Kong Y., Zhuang T., Ding X., Cai S., Ding W., Zhang X., Sun Y., Zhou B., Sun Y., Yang S. (2024). A Five-Plex Hepatic Oncochip Reveals Emt Triplet Correlated with Bap31 in Liver Cancer. Front. Cell Dev. Biol..

[14] Jia C.C., Li G., Jiang R., Liu X., Yuan Q., Le W., Hou Y., Wang B. (2020). B-Cell Receptor-Associated Protein 31 Negatively Regulates the Expression of Monoamine Oxidase A Via R1. Front. Mol. Biosci..

[15] Zhao J., Lv X., Huo Y., Hu X., Li X., Sun S., Zhao X., Kong X., Xu J. (2021). Hepatocyte-Specific Deficiency of Bap31 Amplified Acetaminophen-Induced Hepatotoxicity Via Attenuating Nrf2 Signaling Activation in Mice. Int. J. Mol. Sci..

[16] Wang T., Chen J., Hou Y., Yu Y., Wang B. (2019). Bap31 Deficiency Contributes to the Formation of Amyloid-Beta Plaques in Alzheimer’s Disease by Reducing the Stability of Rtn3. FASEB J..

[17] Liu X., Yuan Q., Li G.X., Jia C.C., Liu J.Y., Yang Y.Q., Wang X.Y., Hou Y., Wang B. (2021). Regulation of Superoxide by Bap31 through Its Effect on P22(Phox) and Keap1/Nrf2/HO-1 Signaling Pathway in Microglia. Oxid. Med. Cell Longev..

[18] Liu X., Jiao K., Jia C.C., Li G.X., Yuan Q., Xu J.K., Hou Y., Wang B. (2019). Bap31 Regulates Irak1-Dependent Neuroinflammation in Microglia. J. Neuroinflamm..

[19] Zhao H., Wang W., Yang Y., Feng C., Lin T., Gong L. (2024). Norepinephrine Attenuates Benzalkonium Chloride-Induced Dry Eye Disease by Regulating the Pink1/Parkin Mitophagy Pathway. Ocul. Immunol. Inflamm..

[20] Zhao Y., Zhu Z., Wang W., Zhang Z., Wen W., Li X. (2024). Pink1/Parkin-Mediated Mitophagy Inhibits the Interferon Response and Promotes Viral Replication During Pseudorabies Virus Infection. Autophagy Rep..

[21] Chidambaram R., Kumar K., Parashar S., Ramachandran G., Chen S., Ferro-Novick S. (2025). Correction: Pink1 Controls Rtn3l-Mediated Er Autophagy by Regulating Peripheral Tubule Junctions. J. Cell Biol..

[22] Krzystek T.J., Banerjee R., Thurston L., Huang J., Swinter K., Rahman S.N., Falzone T.L., Gunawardena S. (2021). Differential Mitochondrial Roles for Alpha-Synuclein in Drp1-Dependent Fission and Pink1/Parkin-Mediated Oxidation. Cell Death Dis..

[23] Seillier M., Pouyet L., N’Guessan P., Nollet M., Capo F., Guillaumond F., Peyta L., Dumas J.F., Varrault A., Bertrand G. (2015). Defects in Mitophagy Promote Redox-Driven Metabolic Syndrome in the Absence of Tp53inp1. EMBO Mol. Med..

[24] Pallanck L., Greenamyre J.T. (2006). Neurodegenerative Disease: Pink, Parkin and the Brain. Nature.

[25] Chen Y., Dorn G.W. (2013). Pink1-Phosphorylated Mitofusin 2 is a Parkin Receptor for Culling Damaged Mitochondria. Science.

[26] Gulati A., Ahn D.H., Suades A., Hult Y., Wolf G., Iwata S., Superti-Furga G., Nomura N., Drew D. (2025). Stepwise Atp Translocation into the Endoplasmic Reticulum by Human Slc35b1. Nature.

[27] Garcia-Gimenez A., Ditcham J.E., Azazi D.M.A., Giotopoulos G., Asby R., Meduri E., Bagri J., Sakakini N., Lopez C.K., Narayan N. (2025). CREBBP Inactivation Sensitizes B Cell Acute Lymphoblastic Leukemia to Ferroptotic Cell Death Upon Bcl2 Inhibition. Nat. Commun..

[28] Clark I.E., Dodson M.W., Jiang C., Cao J.H., Huh J.R., Seol J.H., Yoo S.J., Hay B.A., Guo M. (2006). Drosophila Pink1 is Required for Mitochondrial Function and Interacts Genetically with Parkin. Nature.

[29] Morais V.A., Haddad D., Craessaerts K., De Bock P.J., Swerts J., Vilain S., Aerts L., Overbergh L., Grunewald A., Seibler P. (2014). Pink1 Loss-of-Function Mutations Affect Mitochondrial Complex I Activity Via Ndufa10 Ubiquinone Uncoupling. Science.

[30] Kim H., Kim M., Im S.K., Fang S. (2018). Mouse Cre-Loxp System: General Principles to Determine Tissue-Specific Roles of Target Genes. Lab. Anim. Res..

[31] Shao X., Li M., Shi S., Wu T., Zhang Y., Guo S., Lin H., Qi X. (2025). Neuroprotective Effects of Paeonia Lactiflora Through the Regulation of Gut Dubosiella in an Mptp-Induced Parkinson’s Disease Mouse Model. Am. J. Chin. Med..

[32] More R.V., Patterson R., Pashkovski E., McKinley G.H. (2023). Rod-Climbing Rheometry Revisited. Soft Matter.

[33] Han Y., Li X., Yang L., Zhang D., Li L., Dong X., Li Y., Qun S., Li W. (2022). Ginsenoside Rg1 Attenuates Cerebral Ischemia-Reperfusion Injury Due to Inhibition of Nox2-Mediated Calcium Homeostasis Dysregulation in Mice. J. Ginseng Res..

[34] Dayanc B., Eris S., Gulfirat N.E., Ozden-Yilmaz G., Cakiroglu E., Coskun Deniz O.S., Karakulah G., Erkek-Ozhan S., Senturk S. (2025). Integrative Multi-Omics Identifies Ap-1 Transcription Factor as a Targetable Mediator of Acquired Osimertinib Resistance in Non-Small Cell Lung Cancer. Cell Death Dis..

[35] Wang X., Chen H., Ma X., Liu H., Wu D., Du W., He J., Li S., Chen H., Wu T. (2025). Atrophy of Ventral Diencephalon Is Associated with Freezing of Gait in Parkinson’s Disease: Analysis of Two Cohorts. NPJ Park. Dis..

[36] Chen J., Zhang L., Xie T., Zhang X., Pan C., Sun F., Li W., Sun Z., Dong D. (2025). Nitazoxanide Protects Against Heart Failure with Preserved Ejection and Metabolic Syndrome Induced by High-Fat Diet (Hfd) Plus L-Name “Two-Hit” in Mice. Acta Pharm. Sin. B.

[37] Brustovetsky T., Khanna R., Brustovetsky N. (2025). Collapsin Response Mediator Protein 2 (Crmp2) Modulates Mitochondrial Oxidative Metabolism in Knock-in Ad Mouse Model. Cells.

[38] Korewo-Labelle D., Karnia M.J., Myslinska D., Kaczor J.J. (2025). Impact of Chronic Cold Water Immersion and Vitamin D3 Supplementation on the Hippocampal Metabolism and Oxidative Stress in Rats. Cells.

[39] Yu T., Wang L., Zhang L., Deuster P.A. (2023). Mitochondrial Fission as a Therapeutic Target for Metabolic Diseases: Insights into Antioxidant Strategies. Antioxidants.

[40] Tokuyama T., Yanagi S. (2023). Role of Mitochondrial Dynamics in Heart Diseases. Genes.

[41] Simmen T., Aslan J.E., Blagoveshchenskaya A.D., Thomas L., Wan L., Xiang Y., Feliciangeli S.F., Hung C.H., Crump C.M., Thomas G. (2005). Pacs-2 Controls Endoplasmic Reticulum-Mitochondria Communication and Bid-Mediated Apoptosis. EMBO J..

[42] Machihara K., Namba T. (2019). Bap31 Inhibits Cell Adaptation to ER Stress Conditions, Negatively Regulating Autophagy Induction by Interaction with Stx17. Cells.

[43] Geiger R., Andritschke D., Friebe S., Herzog F., Luisoni S., Heger T., Helenius A. (2011). Bap31 and Bip are Essential for Dislocation of Sv40 from the Endoplasmic Reticulum to the Cytosol. Nat. Cell Biol..

[44] Wakana Y., Takai S., Nakajima K., Tani K., Yamamoto A., Watson P., Stephens D.J., Hauri H.P., Tagaya M. (2008). Bap31 is an Itinerant Protein That Moves Between the Peripheral Endoplasmic Reticulum (Er) and a Juxtanuclear Compartment Related to Er-Associated Degradation. Mol. Biol. Cell.

[45] Yu X., Jia D., Wang Z., Li G., Chen M., Liang Q., Zhou Y., Liu H., Xiao M., Li S. (2021). A Plant Reovirus Hijacks Endoplasmic Reticulum-Associated Degradation Machinery to Promote Efficient Viral Transmission by Its Planthopper Vector Under High Temperature Conditions. PLoS Pathog..

[46] Kuijpers M., van Dis V., Haasdijk E.D., Harterink M., Vocking K., Post J.A., Scheper W., Hoogenraad C.C., Jaarsma D. (2013). Amyotrophic Lateral Sclerosis (Als)-Associated Vapb-P56s Inclusions Represent an Er Quality Control Compartment. Acta Neuropathol. Commun..

[47] Iida R., Ueki M., Yasuda T. (2015). Identification of Interacting Partners of Human Mpv17-like Protein with a Mitigating Effect of Mitochondrial Dysfunction Through mtdna Damage. Free Radic. Biol. Med..

[48] Du Y., Zhu P., Wang X., Mu M., Li H., Gao Y., Qin X., Wang Y., Zhang Z., Qu G. (2020). Pirfenidone Alleviates Lipopolysaccharide-Induced lung Injury by Accentuating Bap31 Regulation of Er Stress and Mitochondrial Injury. J. Autoimmun..

[49] Plaas M., Karis A., Innos J., Rebane E., Baekelandt V., Vaarmann A., Luuk H., Vasar E., Koks S. (2008). Alpha-Synuclein A30P Point-Mutation Generates Age-Dependent Nigrostriatal Deficiency in Mice. J. Physiol. Pharmacol..

[50] Schalkwyk L.C., Fernandes C., Nash M.W., Kurrikoff K., Vasar E., Koks S. (2007). Interpretation of Knockout Experiments: The Congenic Footprint. Genes Brain Behav..

[51] Koks S., Soomets U., Paya-Cano J.L., Fernandes C., Luuk H., Plaas M., Terasmaa A., Tillmann V., Noormets K., Vasar E. (2009). Wfs1 Gene Deletion Causes Growth Retardation in Mice and Interferes with the Growth Hormone Pathway. Physiol. Genom..

[52] Koks S., Mannisto P.T., Bourin M., Shlik J., Vasar V., Vasar E. (2000). Cholecystokinin-Induced Anxiety in Rats: Relevance of Pre-Experimental Stress and Seasonal Variations. J. Psychiatry Neurosci..

[53] Raza A., Hoque A., Luwor R., Escalona R.M., Kelly J., Sharma R., Charchar F., Chu S., Short M.K., Jubinsky P.T. (2025). Enhanced Expression of Mitochondrial Magmas Protein in Ovarian Carcinomas: Magmas Inhibition Facilitates Antitumour Effects, Signifying a Novel Approach for Ovarian Cancer Treatment. Cells.

[54] Qin D., Hu W., Guo Y., Cheng R., Hao F., Zhao B. (2025). Baicalein Based Nano-Delivery System Restores Mitochondrial Homeostasis Through Ppar Signaling Pathway to Promote Wound Healing in Diabetes. J. Nanobiotechnol..

[55] Li Z., Okamoto K., Hayashi Y., Sheng M. (2004). The Importance of Dendritic Mitochondria in the Morphogenesis and Plasticity of Spines and Synapses. Cell.

[56] Sommers O., Tomsine R.A., Khacho M. (2024). Mitochondrial Dynamics Drive Muscle Stem Cell Progression from Quiescence to Myogenic Differentiation. Cells.

[57] Wang G., Li Z., Han W., Tian Q., Liu C., Jiang S., Xiang X., Zhao X., Wang L., Liao J. (2025). Itaconate Promotes Mitophagy to Inhibit Neuronal Ferroptosis After Subarachnoid Hemorrhage. Apoptosis.

[58] Park J., Lee S.B., Lee S., Kim Y., Song S., Kim S., Bae E., Kim J., Shong M., Kim J.M. (2006). Mitochondrial Dysfunction in Drosophila Pink1 Mutants is Complemented by Parkin. Nature.

[59] Pattingre S., Turtoi A. (2022). Bag Family Members as Mitophagy Regulators in Mammals. Cells.

[60] Koks S., Nikopensius T., Koido K., Maron E., Altmae S., Heinaste E., Vabrit K., Tammekivi V., Hallast P., Kurg A. (2006). Analysis of Snp Profiles in Patients with Major Depressive Disorder. Int. J. Neuropsychopharmacol..

