# Supplementary material for: BAP31 Modulates Mitochondrial Homeostasis Through PINK1/Parkin Pathway in MPTP Parkinsonism Mouse Models
Source: Cells. 2026 Jan 12;15(2):137. doi: 10.3390/cells15020137 (PMC12839962; doi:10.3390/cells15020137)
Supplement: Supplementary file 1 [file cells-15-00137-s001.zip › cells-4053188-supplementary.pdf]

Gene	left	Right
BAP31	AGAAGGCTCGAAGCACGTTG	AGGGATGGGAGCTTGTTTCCTTT
PINK1	ATCTGGTTCAGCAGGGCATT	AGGGACAGCCATCTGAGTCC
Parkin	TAGCTTTGCACCTGATCGCA	CTAAGCAAATCACGTGGCGG
DJ-1	GAGGCGAGCTGGGATTAAGG	ACCACATCACGGCTACACTG
LRRK2	GCCATGCACAGATATTCAGCC	CATGGGCATGCTTCTGCATC
GBA1	GCCTCCCAGAAGAAGACACC	ATATCCCCTGGCTGACCCTT
SCNA	GCAAGAATGAAGAAGGAGCCC	AGCCTCATTGTCAGGATCCAC
NPC1	ATGTACAATGCCTGCCGTGA	ACACAAAGTACCGCCTTCTGT
GAA	TGTGGCTCGACATGAACGAA	AGGTAGGGCAGAAGGGCATA
APP	CCTCCGTGTGATCTACGAGC	GAACCTGGTCGAGTGGTCAG
MAPT	ACACGGAGATCCCAGAAGGA	CTTTAGGTCTGGCATGGGCA
AGL	CCTGTCACTGGGTTCTGTCC	TCGTACGAGACTGCCACAAC
ATP7B	TGCTGTCTGTGCAGAAGAGG	GCCCTCGATAGAGCTGACAC
PSEN1	GAATGACAGCCAAGAACGGC	GATCGAGTGCAGGGCTCTTT
SQSTM1	GGACCCATCTACAGAGGCTG	ACAATGGTGGAGGGTGCTTC
APOE	CCTGAACCGCTTCTGGGATT	TGCCTTGTACACAGCTAGGC
GBE1	TCACCACGGAATGGGTCAAG	TAGTCAAAACCACCGCCTCC
LMNA	GGCGGTAGAGGAAGTCGATG	GCTCTTCTCCATCCTCGTCG
SMPD1	ACTACCCCGGAAGCTCTCAT	ATGCGGTAGACCAGGTTGTG
DYNC1H1	GGCCCACATAATTGACCCCA	GCGTCGCGTATTTCAAGTCC

Figure S1 The sequence encoding of mRNA

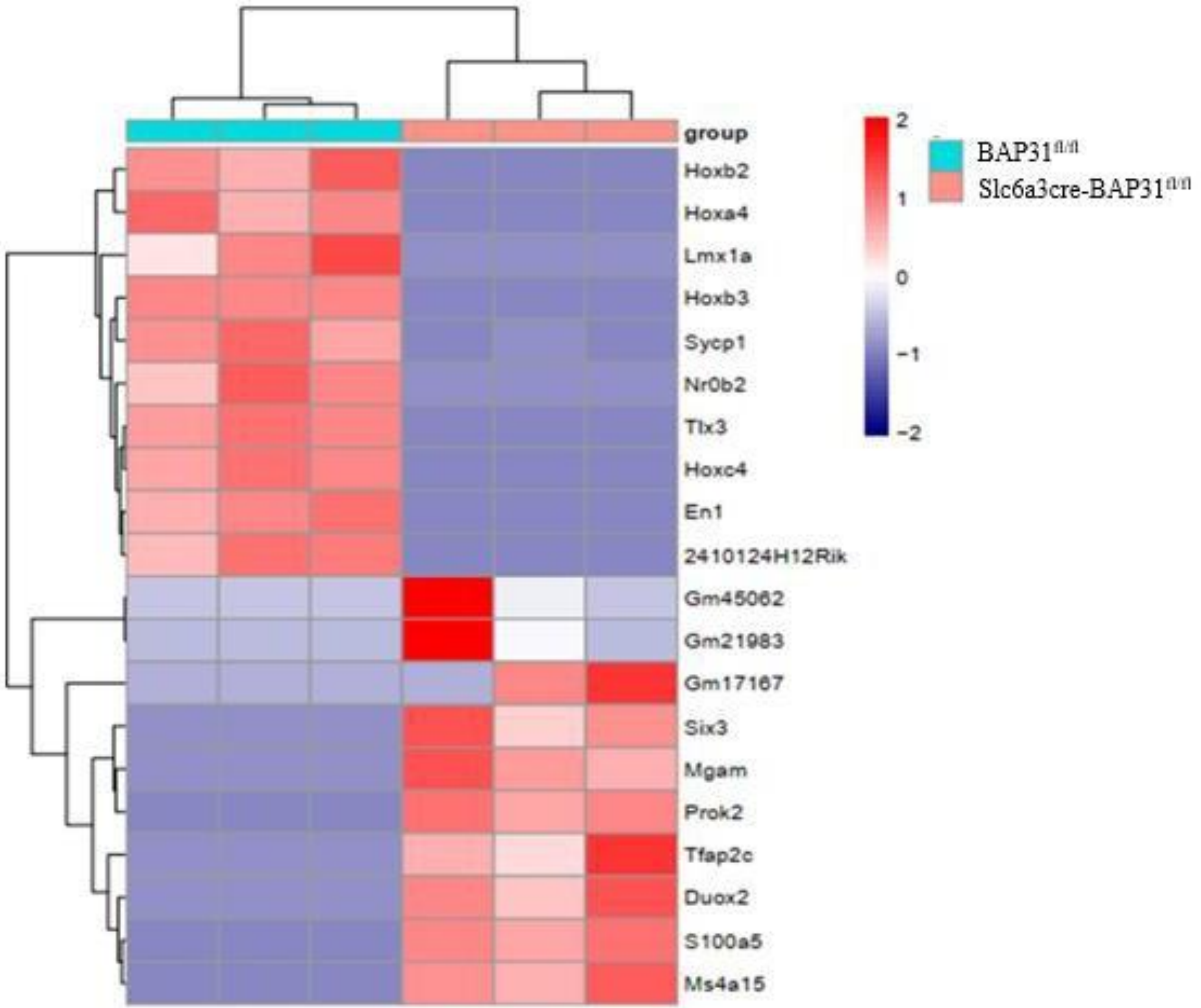

Figure S2 Shows the heat map analysis of differentially expressed genes in the brain of BAP31$^{fl/fl}$ and Scl6a3 cre-BAP31$^{fl/fl}$ mice.

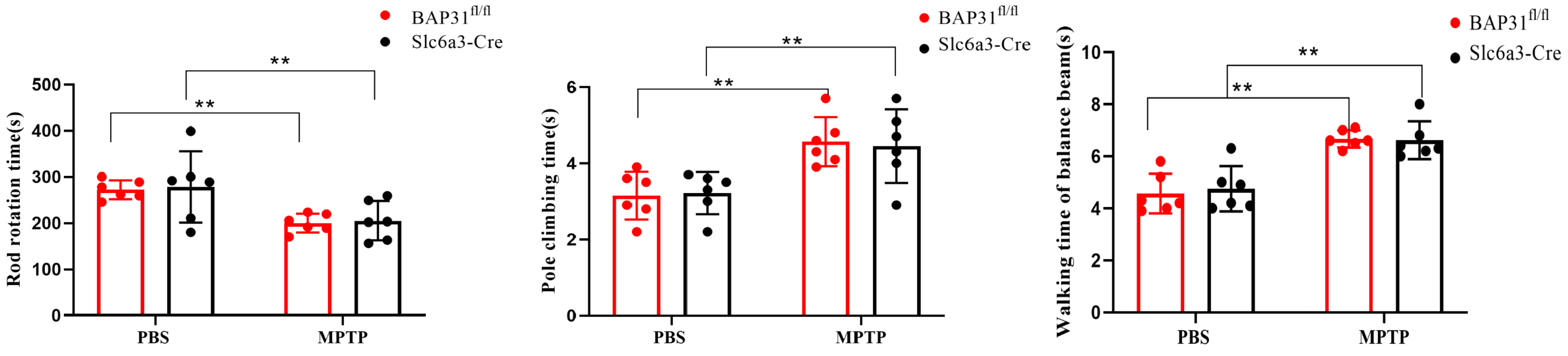

Figure S3 Behavioral results (Rotating rod experiment,Pole climbing experiment and Beam walking experiment) of 6-month-old Slc6a3-Cre and BAP31$^{fl/fl}$ mice injected with PBS or MPTP. (Experimental groups: BAP31$^{fl/fl}$ mice, n = 6; Slc6a3cre-BAP31$^{fl/fl}$ mice, n = 6; BAP31$^{fl/fl}$ mice injected with MPTP, n = 6; and Slc6a3cre-BAP31$^{fl/fl}$ mice injected with MPTP, n = 6).

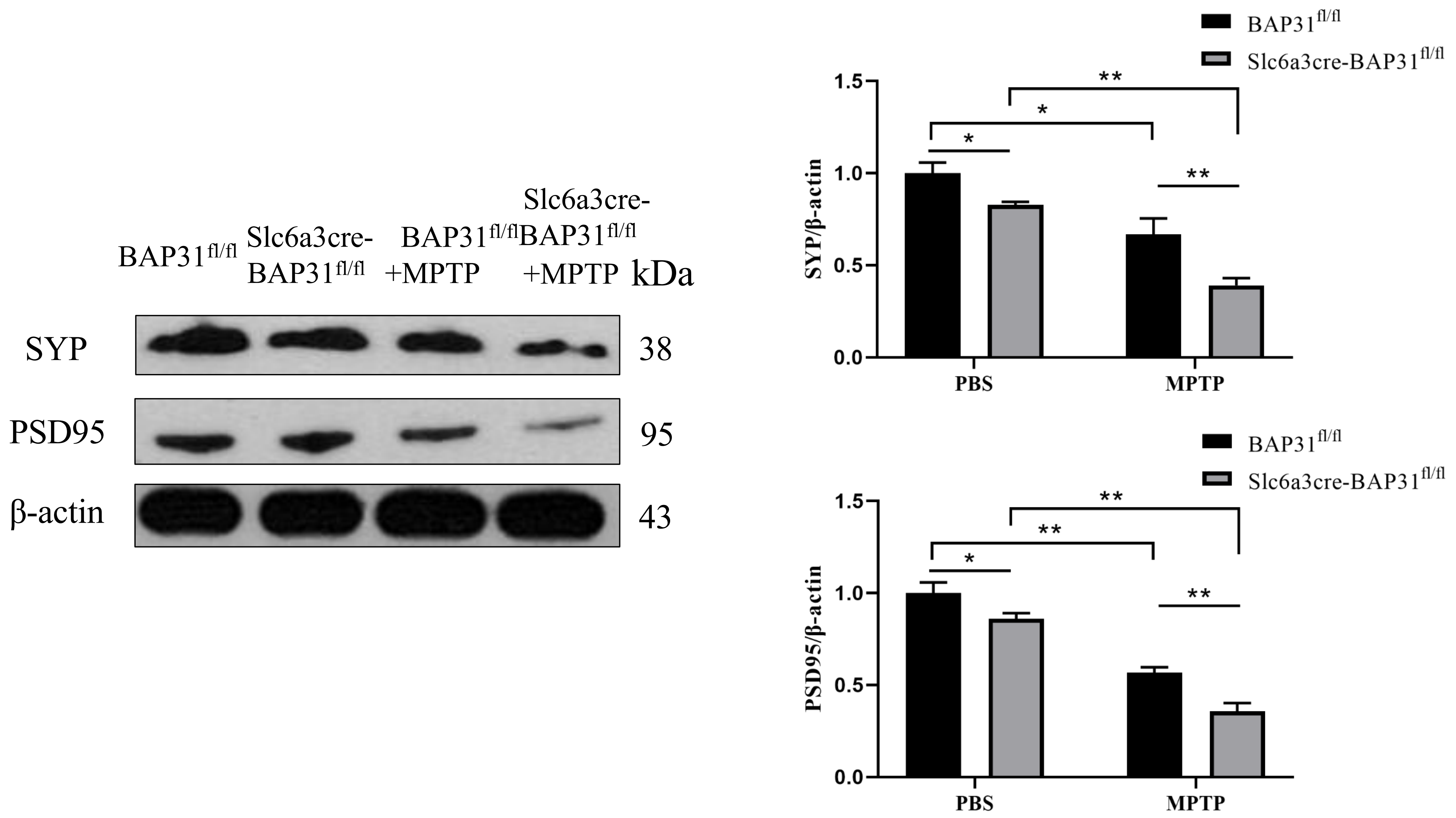

Figure S4 The content of SYP, PSD95 in the midbrain and striatum were detected by WB. (Experimental groups: BAP31$^{fl/fl}$ mice, n = 3; Slc6a3cre-BAP31$^{fl/fl}$ mice, n = 3; BAP31$^{fl/fl}$ mice injected with MPTP, n = 3; and Slc6a3cre-BAP31$^{fl/fl}$ mice injected with MPTP, n = 3).

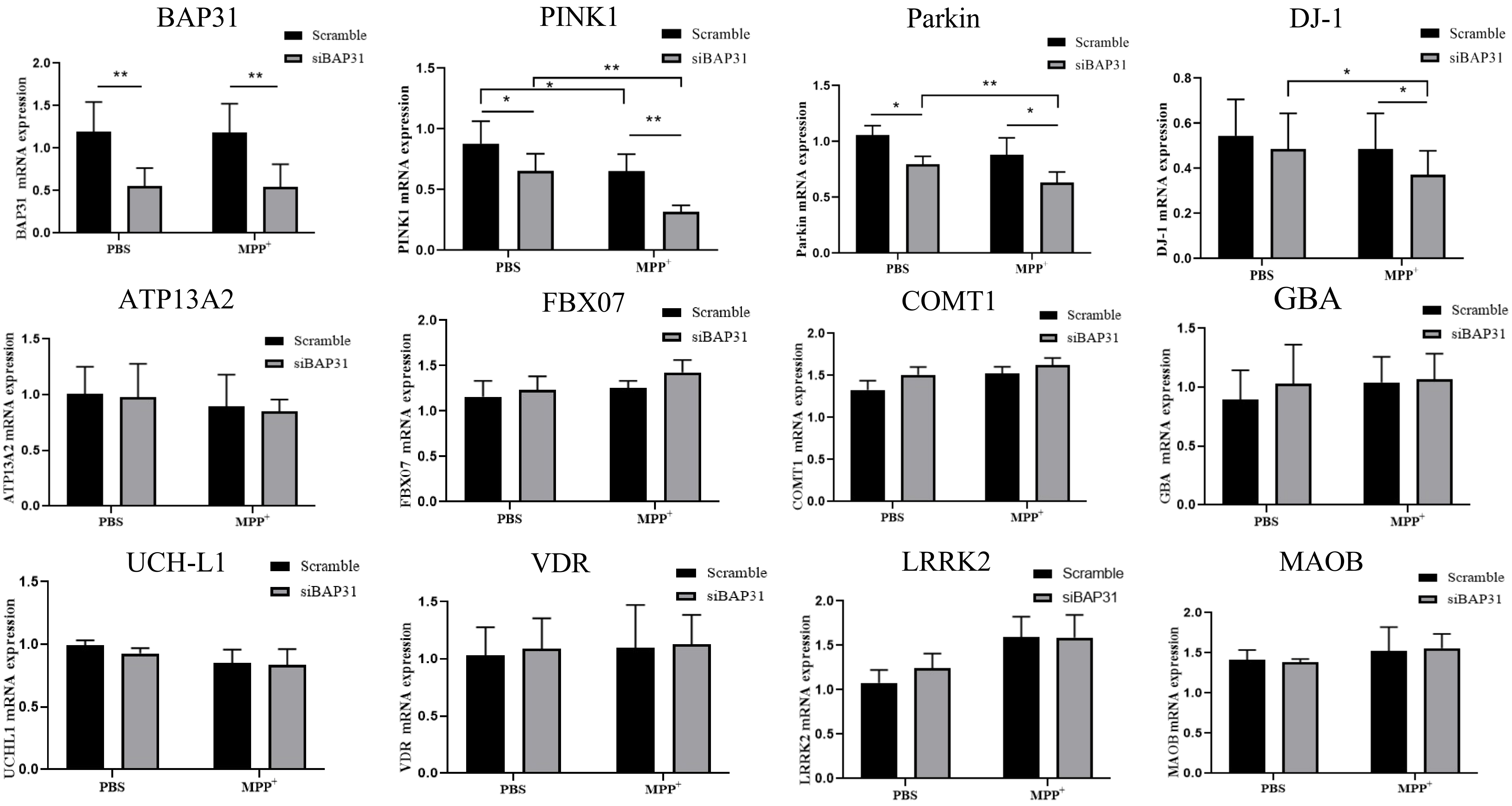

Figure S5 The mRNA levels of each protein in the SH-SY5Y cells were analyzed by qPCR.
